# Gender differences in under-reporting hiring discrimination in Korea: a machine learning approach

**DOI:** 10.4178/epih.e2021099

**Published:** 2021-11-17

**Authors:** Jaehong Yoon, Ji-Hwan Kim, Yeonseung Chung, Jinsu Park, Glorian Sorensen, Seung-Sup Kim

**Affiliations:** 1 Department of Public Health Sciences, Graduate School of Korea University, Seoul, Korea; 2 Department of Mathematical Sciences, Korea Advanced Institute of Science and Technology, Daejeon, Korea; 3 Department of Social and Behavioral Sciences, Harvard T.H. Chan School of Public Health, Boston, MA, USA; 4 Center for Community-Based Research, Dana-Farber Cancer Institute, Boston, MA, USA; 5 Interdisciplinary Program in Precision Public Health, Korea University, Seoul, Korea

**Keywords:** Social discrimination, Social epidemiology, Machine learning

## Abstract

**OBJECTIVES:**

This study was conducted to examine gender differences in under-reporting hiring discrimination by building a prediction model for workers who responded “not applicable (NA)” to a question about hiring discrimination despite being eligible to answer.

**METHODS:**

Using data from 3,576 wage workers in the seventh wave (2004) of the Korea Labor and Income Panel Study, we trained and tested 9 machine learning algorithms using “yes” or “no” responses regarding the lifetime experience of hiring discrimination. We then applied the best-performing model to estimate the prevalence of experiencing hiring discrimination among those who answered “NA.” Under-reporting of hiring discrimination was calculated by comparing the prevalence of hiring discrimination between the “yes” or “no” group and the “NA” group.

**RESULTS:**

Based on the predictions from the random forest model, we found that 58.8% of the “NA” group were predicted to have experienced hiring discrimination, while 19.7% of the “yes” or “no” group reported hiring discrimination. Among the “NA” group, the predicted prevalence of hiring discrimination for men and women was 45.3% and 84.8%, respectively.

**CONCLUSIONS:**

This study introduces a methodological strategy for epidemiologic studies to address the under-reporting of discrimination by applying machine learning algorithms.

## INTRODUCTION

Discrimination harms people’s lives [1]. It can deprive them of socioeconomic opportunities in daily life (e.g., education, employment, and income), and these disadvantages can accumulate over the life-course [2-4]. Furthermore, the experience of discrimination can exert adverse health consequences [5]. Mounting evidence implicates discrimination in an increased risk of hypertension [6,7], cardiovascular disease [8,9], depressive symptoms [10-12], and suicide [13-15]. Indeed, a meta-analysis of 333 articles published between 1983 and 2013 found concrete epidemiologic evidence on the health impacts of discrimination [16].

However, an important issue in the field is the potential underreporting of discriminatory experiences. Several factors could lead to the under-reporting of discrimination [3,17]. One factor is social desirability, which is the tendency for survey participants to answer questions in a way that they deem socially acceptable [3]. Another relevant factor is “internalized oppression,” in which people in subordinate groups accept their social status and internalize negative attitudes towards them, thus perceiving their mistreatment experience as being deserved [3].

Previous studies have sought to understand the mechanism of reporting discrimination, especially in socially vulnerable groups. For example, in a study of 400 people living in a Boston suburb, women tended to deny their personal experience of gender discrimination despite knowing that women workers generally did not receive the opportunities and rewards they deserved [18]. Using a dataset from Korea, Kim et al. [19] compared the predicted logit score of discrimination between genders and found that women workers tended to under-report their discriminatory experiences. A body of literature has also identified the “personal/group discrimination discrepancy,” which is the tendency for people to under-report their personal experiences of discrimination while still acknowledging discrimination against their group [20,21].

Machine learning offers effective approaches to improving predictive performance in various fields, including social epidemiology [22-24]. Machine learning algorithms have facilitated research on the role of social factors in predicting individual health conditions. These factors include community-level socioeconomic status (SES) [25,26], individual-level SES [23,27], language skill [28], and insurance coverage [23,28]. For example, a recent study of 66 low-income and middle-income countries explored the ability of SES indicators, including household wealth, educational attainment, and occupation, to predict women’s height, which could be an indicator of human welfare at the population level [27]. Thus, machine learning may facilitate attaining a similarly improved understanding of discrimination.

This study sought to investigate the following research purposes using a nationally representative dataset in Korea, with the following goals: (1) to apply 9 machine learning algorithms to build a prediction model for the experience of hiring discrimination among wage workers; (2) to predict whether workers who responded “not applicable (NA)” actually experienced hiring discrimination using the best prediction model; (3) to assess underreporting of discrimination by comparing the prevalence of hiring discrimination between training sample (“yes” or “no” group) and the prediction sample (“NA” group); and (4) to examine gender differences in the under-reporting of discrimination.

## MATERIALS ANS METHODS

### Study population

**Figure 1.**
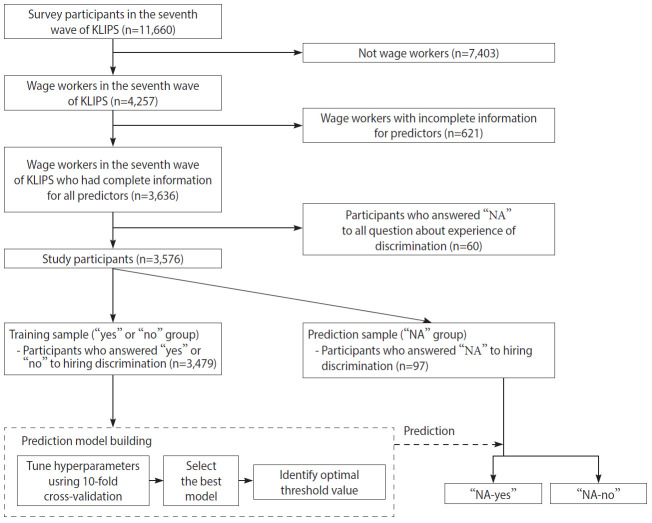
Flow chart of data analysis. KLIPS, Korea Labor and Income Panel Study; NA, not applicable.

This study analyzed a nationally representative longitudinal dataset obtained from the Korean Labor and Income Panel Study (KLIPS), launched in 1998. Five thousand households (13,321 respondents) participated in the first wave of the KLIPS. KLIPS data from the first (1998) through 22nd (2019) waves are publicly available (https://www.kli.re.kr). We utilized data from the seventh wave (2004), the only one in which the KLIPS measured the experience of discrimination. It provided a unique chance to investigate the behavior of responding “NA” among those eligible to answer a question about hiring discrimination. Figure 1 shows the flow chart of data analysis. Our analyses included wage workers at the time of the survey (n=4,257) to ensure that all survey participants were eligible to answer the question about the experience of hiring discrimination. The exclusion criteria were: participants with missing information on any of the variables used in the prediction model (n=621), and those who responded NA to all questions about discrimination (n=60) because the experience of discrimination in other situations provided critical information to improve the performance of the predictive models. The final sample size was 3,576 (Figure 1).

### Target variable

Hiring discrimination was measured by the question, “Have you ever experienced discrimination in getting hired?” Respondents could answer “no,” “yes,” or “NA”. All included individuals were eligible to answer either “yes” or “no” to the question because they were wage workers at the time of the survey. However, 97 participants (2.7%) responded “NA” to the experience of hiring discrimination. Machine learning models were built to predict whether those who answered “NA” (prediction sample, n=97) experienced hiring discrimination, using the information from those who answered “yes” (n=686) and “no” (n=2,793; training sample, n=3,479) (Figure 1).

### Predictors

We selected variables to be included in the prediction model by referring to the literature on discrimination in Korea [4,29]. Gender, age, education level, marital status, employment status, equivalized household income, birth region, self-rated health condition, disability, residential area, and experience of discrimination in other situations were included as predictors. All predictors used in the analyses were obtained from the seventh wave of the survey, except for information on disability (only available in wave 9). Age was categorized into six groups (16-24, 25-34, 35-44, 45-54, 55-64, ≥ 65 years old). Education level was measured and classified into 3 categories: middle school graduate or less, high school graduate, and college graduate or more. Marital status was assessed in 3 categories: never married, currently married, and previously married. Employment status was assessed using 2 questions about status of workers (i.e., permanent, temporary, daily) and type of working hours (i.e., full-time vs. part-time). Full-time permanent workers were defined as the permanent group, whereas all other workers were classified as the non-permanent group. Equivalized household income was assessed by dividing the total household income by the square root of the number of household members, and participants were classified into 4 categories based on the quartiles. Birth region was separated into Jeolla Province and other regions considering the stigma that has existed against people born in Jeolla Province [30]. Self-rated health condition was assessed on a 5-point scale in response to the question, “How would you rate your health?” Participants could choose a response from “very good” (score 1) to “very poor” (score 5). Considering the small number of very poor and poor responses, the 2 groups were merged into poor for analysis. Disability was measured by the yes/no question, “Do you have any impairment or disability?” Residential area was measured at the metropolitan and provincial levels (i.e., Seoul, Busan, Daegu, Daejeon, Incheon, Gwangju, Ulsan, Gyeonggi Province, Gangwon Province, Chungcheongbuk Province, Chungcheongnam Province, Jeollabuk Province, Jeollanam Province, Gyeongsangbuk Province, Gyeongsangnam Province). Finally, experiences of discrimination in other situations were measured using a modified version of the “Experience of Discrimination” questionnaire. Participants could answer “no,” “yes,” or “NA” for their experiences of discrimination in each of the following seven situations: receiving income, training, getting promoted, being fired, obtaining higher education, at home, and general social activities.

### Building a prediction model

We considered 9 machine learning algorithms to build a prediction model for the experience of hiring discrimination among training sample (“yes” or “no” group): logistic regression, random forest, penalized logistic regression (ridge, lasso, and elastic net), k-nearest neighbor, support vector machine (polynomial and radial basis kernel functions), and a single-layer artificial neural network. Supplementary Material 1 details the machine learning algorithms and tuning parameters. We conducted 10-fold cross-validation to identify the optimal tuning parameters for each of the 9 algorithms. For cross-validation, we split the data into 10 folds such that the experience of hiring discrimination was equally distributed in each fold to prevent an imbalanced distribution of the target variable.

The predictive performance of machine learning algorithms was assessed using the area under the curve (AUC) of the receiver operating characteristic (ROC) curve. We selected the algorithm with the largest AUC as the best-performing algorithm and determined the optimal threshold for the binary prediction based on the ROC curve from the best-performing algorithm such that the sum of sensitivity and specificity was maximized. We then predicted the binary response of experiencing hiring discrimination among those in the prediction sample, and individuals were classified into 2 groups: “NA-yes” (> threshold) or “NA-no” (≤ threshold).

Finally, we applied a modified Poisson regression model with a robust error variance to investigate the under-reporting of discrimination by comparing the prevalence of hiring discrimination between training sample and prediction sample. The odds ratio estimated in logistic regression can over-estimate the prevalence ratio (PR), given the high outcome prevalence (> 10%) [31]. Results from the association analyses are presented as PRs with 95% confidence intervals (CIs). All machine learning algorithms were estimated from the tidymodels package in R version 4.0.2 (R Core Team, Vienna, Austria).

### Sensitivity analyses

We checked whether our results were robust to: (1) the exclusion or inclusion of people who answered “NA” to experiences of discrimination in all 7 situations other than hiring (n=60), and (2) different choices of the probability threshold used to classify the experience of hiring discrimination. These aspects were considered separately at first and later considered together. Therefore, we conducted 3 sensitivity analyses (Supplementary Material 2).

### Ethics statement

This study was exempt from review by the Institutional Review Board of the Office of Human Research Administration at Korea University (KUIRB-2021-0049-01).

## RESULTS

Table 1 shows the distribution of the study population and the prevalence of hiring discrimination by different categories for each predictor. The overall prevalence of hiring discrimination was 19.2% (n = 686). The prevalence of hiring discrimination tended to be higher among participants who were older, had a lower education level, non-permanent employment status, lower household income, poor self-rated health conditions, or experiences of discrimination in other situations.

**Figure 2.**
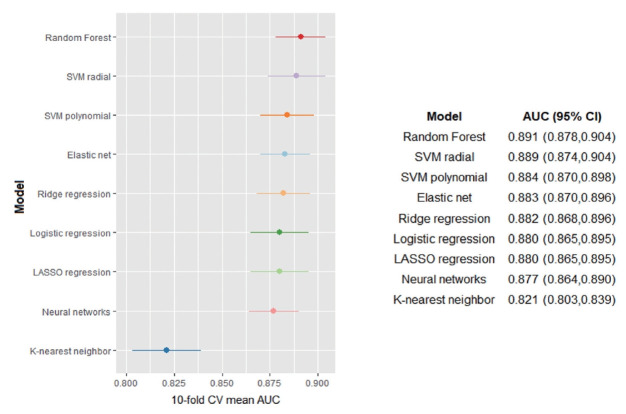
Cross-validated performance of the machine learning algorithms according to the area under the curve (AUC). CV, cross-validation; CI, confidence interval.

Figure 2 shows the cross-validated AUC for each of the 9 machine learning algorithms; the corresponding ROC curves are depicted in Supplementary Material 3. The AUCs for each algorithm ranged from 0.821 to 0.891; the random forest algorithm performed the best among all algorithms, showing the highest AUC (AUC, 0.891; 95% CI, 0.878 to 0.904). The AUC of other machine learning algorithms, except for k-nearest neighbor algorithms, was similar to that of the random forest model. The variable importance scores of predictors for classifying hiring discrimination in the random forest algorithm (best-performing algorithm) are illustrated in Supplementary Material 4. Experience of discrimination in receiving income had the highest score, followed by discriminatory experiences in general social activities and in getting promoted. In the random forest algorithm, the optimal threshold value was determined as 0.268 (Supplementary Material 5).

Based on the prediction from the random forest algorithms, we compared the prevalence of hiring discrimination between the training sample (“yes” or “no” group) and prediction sample (“NA” group) to investigate the under-reporting of hiring discrimination (Table 2). We found that 58.8% of the “NA” group were predicted to experience hiring discrimination, while 19.7% of the “yes” or “no” group reported hiring discrimination. This finding shows that the predicted prevalence of hiring discrimination among workers who responded “NA” is about 3 times higher than those among workers who responded “yes” or “no” (PR, 2.98; 95% CI, 2.49 to 3.57). Combining the observed prevalence from the training sample (“yes” or “no” group) and the predicted prevalence from the prediction sample (“NA” group), we estimated that the prevalence of hiring discrimination among the total population could be 20.8% (n=743) if we count NA-yes as those who actually experienced discrimination in addition to the “yes” group. Since the prevalence of hiring discrimination among the “yes” or “no” group was 19.7% (n=686), this finding could imply that under-reporting has occurred during the process of reporting discriminatory experiences.

We also found a gender difference in under-reporting of hiring discrimination (Table 2). The predicted prevalence of hiring discrimination among the men and women prediction sample (“NA” group) was 45.3% and 84.8%, respectively, whereas the observed prevalence among the men and women training sample (“yes” or “no” group) was 18.8% and 21.1%, respectively. Although the predicted prevalence of hiring discrimination was higher than the observed prevalence among men and women workers, under-reporting of hiring discrimination was more substantial among women workers (PR, 4.02; 95% CI, 3.37 to 4.79) than among men workers (PR, 2.41; 95% CI, 1.82 to 3.20). The estimated actual prevalence of hiring discrimination was 22.6% among all women workers, while the estimated actual prevalence was 19.6% among all men workers. Therefore, the prevalence of hiring discrimination could be underestimated without considering the NA response resulting from under-reporting, especially among women workers.

In sensitivity analyses, we evaluated the robustness of our results, considering the selection criteria for the probability threshold used to classify the experience of hiring discrimination and the inclusion criteria for the study population (Supplementary Material 2). First, we applied a different selection criterion when determining a probability threshold for the classification (Supplementary Material 6). The results of sensitivity analysis 1 were consistent with those of the main analyses and showed that women workers were more likely to under-report hiring discrimination (Supplementary Material 7). Second, we included participants who responded “NA” to all questions about the experience of discrimination in the analysis (sensitivity analysis 2 and sensitivity analysis 3), and the results of the cross-validated AUC were similar to those of the main analyses (Supplementary Materials 8 and 9). Gender differences in the under-reporting of hiring discrimination (Supplementary Materials 10 and 11) also showed similar trends as the main analyses.

## DISCUSSION

The results of this study demonstrate the potential to build a performative prediction model for the experience of discrimination using covariates, including demographic and SES information. We found that the predicted prevalence of hiring discrimination in the prediction sample (“NA” group) was higher than those observed in the training sample (“yes” or “no” group), which could imply under-reporting of hiring discrimination among Korean workers. Furthermore, we found that the under-reporting of hiring discrimination differed by gender.

These results are consistent with findings from previous studies investigating the personal-group discrimination discrepancy [20,21]. The authors in those studies postulated that vulnerable groups might tend to conceal their personal experiences of discrimination even though they recognize that their group experiences discrimination. Furthermore, Operario & Fiske [32] used a quantitative study design to examine the personal-group discrimination discrepancy across ethnic groups and found a higher likelihood of the personal-group discrimination discrepancy among ethnic minorities than among White people.

Applying machine learning algorithms, we found that there might be substantial under-reporting of discriminatory experience in hiring situations both among men and women who responded “NA” to the question. Previous studies have indicated that asking sensitive questions in a survey could lead to under-reporting of respondents’ experiences [33,34]. For example, it was argued that survey respondents might misreport their responses to a sensitive question, even after they decide to answer it, to avoid embarrassing themselves [34,35]. Furthermore, people who have experienced hiring discrimination might avoid the question to conceal their discriminatory experiences so that their response would be recognized as socially desirable by the interviewer [34,36]. This under-reporting may have been an issue, especially with the KLIPS, in which data were collected through in-person interviews. Additionally, some victims of discrimination might have felt that it was painful to acknowledge that they had been victimized by unfair treatment, which could be a reason for under-reporting their experiences [35].

Notably, we found that under-reporting of hiring discrimination was more prevalent among women than among men. Previous studies have provided several possible explanations for the observed gender differences in the prevalence of the “NA-yes” group. For example, there may be gender differences in the extent to which internalized oppression is experienced among those who answer “NA” to the hiring discrimination question. People from vulnerable groups may internalize negative attitudes against themselves and perceive their discriminatory experience as nondiscriminatory [3,17]. Previous studies also indicated that socially vulnerable groups, including women, are more likely to internalize and accept unfair treatment against them [37,38], which might lead them not to report their experiences of discrimination.

The gender difference in under-reporting hiring discrimination observed in this study could be a public health concern. Although previous studies have indicated that not disclosing a violent event to others can negatively influence the victims’ health [39-41], few studies have examined the underestimating the health impacts of discrimination due to under-reporting. As a post-hoc analysis, we examined the association between hiring discrimination and self-rated health after dividing the responses to hiring discrimination among the entire population into 4 groups: “no,” “yes,” “NA-no,” and “NA-yes” (Supplementary Material 12). The “NA-yes” group was more likely to have poor self-rated health than the “no” group, and this association was statistically significant among both men and women workers. These results should be interpreted cautiously because we could not adjust for confounding variables due to the small sample size. Therefore, future studies with a larger sample are necessary to assess the potential underestimation in the association between discrimination and health.

This study has several limitations. First, people who did not have a job in the seventh wave of the KLIPS were excluded from the analyses because we could not determine their eligibility to answer the question of hiring discrimination. This exclusion may have led to the loss of information on people who experienced severe hiring discrimination and as a result were unemployed, which might have improved the performance of the prediction models. Second, unmeasured predictors may have contributed to increasing the predictive power of our models, although our prediction model showed relatively high performance (AUC of the random forest model: 0.891). For example, information on physical appearance and sexual orientation were not measured in the KLIPS, although these variables might be a source of discriminatory experience in getting hired. Third, a relatively small sample (n=3,479) was used to build a prediction model of hiring discrimination. A previous study indicated that machine learning algorithms could need a larger sample than that is required for logistic regression to avoid overfitting [42]. Therefore, future studies need to analyze a larger sample size to build prediction models for hiring discrimination. Fourth, our results might not be generalizable to other populations considering the time-point when data analyzed in our study was collected. For example, the Korean government has applied blind recruitment to national competency standards since 2017 [43]. The probability of hiring discrimination in 2004 could be different from that in 2021 when blind recruitment is applied. However, we could not find other datasets available for replicating our hypothesis. Future studies should investigate whether the gender difference in under-reporting of hiring discrimination can be observed in other populations.

To the best of our knowledge, this is the first study to assess the magnitude of under-reporting of hiring discrimination using machine learning algorithms. Additionally, by applying machine learning algorithms, this study showed overwhelming superiority over a previous study investigating the predictive probability of having experienced hiring discrimination based on the same dataset [20]. The previous study did not consider multiple models for the prediction, did not assess the performance of their prediction model by using metrics such as the AUC, and did not estimate the predictive probability of hiring discrimination for each individual.

This study found that there could be a potential underestimation of hiring discrimination among Korean workers due to under-reporting. Furthermore, women workers were more likely to under-report their experience of hiring discrimination by answering “NA.” This study introduces a strategy to apply machine learning algorithms for social epidemiology studies addressing the under-reporting of discriminatory experiences.

## Figures and Tables

**Table 1.** Distribution of the study population and prevalence of hiring discrimination by predictors among wage workers in Korea

Characteristics		Distribution	Prevalence of hiring discrimination	p-value^1^
Overall		3,576 (100)	686 (19.2)	
Gender				0.131
	Men	2,165 (60.5)	395 (18.2)	
	Women	1,411 (39.5)	291 (20.6)	
Age (yr)				<0.001
	16-24	277 (7.7)	54 (19.5)	
	25-34	1,124 (31.4)	185 (16.5)	
	35-44	1,025 (28.7)	178 (17.4)	
	45-54	744 (20.8)	147 (19.8)	
	55-64	306 (8.6)	88 (28.8)	
	≥65	100 (2.8)	34 (34.0)	
Education				<0.001
	Middle school graduate or less	851 (23.8)	262 (30.8)	
	High school graduate	1,445 (40.4)	270 (18.7)	
	College graduate or more	1,280 (35.8)	154 (12.0)	
Marital status				<0.001
	Never married	869 (24.3)	186 (21.4)	
	Currently married	2,501 (69.9)	432 (17.3)	
	Previously married	206 (5.8)	68 (33.0)	
Employment status				<0.001
	Permanent	2,728 (76.3)	434 (15.9)	
	Non-permanent	848 (23.7)	252 (29.7)	
Household income				<0.001
	Less than Q1	554 (15.5)	164 (29.6)	
	Q1-Q2	909 (25.4)	232 (25.5)	
	Q2-Q3	1,005 (28.1)	165 (16.4)	
	>Q3	1,108 (31.0)	125 (11.3)	
Birth region				0.082
	Other regions	2,892 (80.9)	574 (19.8)	
	Jeolla Province	684 (19.1)	112 (16.4)	
Self-rated health conditions				<0.001
	Very good	161 (4.5)	18 (11.2)	
	Good	2,061 (57.6)	362 (17.6)	
	Fair	1,115 (31.2)	236 (21.2)	
	Poor and very poor	239 (6.7)	70 (29.3)	
Having a disability				<0.001
	No	3,484 (97.4)	651 (18.7)	
	Yes	92 (2.6)	35 (38.0)	
Residential area				<0.001
	Seoul	811 (22.7)	100 (12.3)	
	Busan	354 (9.9)	123 (34.7)	
	Daegu	212 (5.9)	60 (28.3)	
	Daejeon	112 (3.1)	22 (19.6)	
	Incheon	258 (7.2)	30 (11.6)	
	Gwangju	98 (2.7)	21 (21.4)	
	Ulsan	115 (3.2)	31 (27.0)	
	Gyeonggi Province	769 (21.5)	119 (15.5)	
	Gangwon Province	40 (1.1)	9 (22.5)	
	Chungcheongbuk Province	80 (2.2)	18 (22.5)	
	Chungcheongnam Province	101 (2.8)	18 (17.8)	
	Jeollabuk Province	140 (3.9)	24 (17.1)	
	Jeollanam Province	80 (2.2)	10 (12.5)	
	Gyeongsangbuk Province	157 (4.4)	42 (26.8)	
	Gyeongsangnam Province	249 (7.0)	59 (23.7)	
Discriminatory experience				<0.001
	Income			
	No	2,980 (83.3)	260 (8.7)	
	Yes	545 (15.2)	418 (76.7)	
	Not applicable	51 (1.4)	8 (15.7)	
	Training			<0.001
	No	3,136 (87.7)	470 (15.0)	
	Yes	71 (2.0)	53 (74.6)	
	Not applicable	369 (10.3)	163 (44.2)	
	Promotion			<0.001
	No	2,924 (81.8)	398 (13.6)	
	Yes	209 (5.8)	108 (51.7)	
	Not applicable	443 (12.4)	180 (40.6)	
	Fired			<0.001
	No	3,098 (86.6)	501 (16.2)	
	Yes	65 (1.8)	53 (81.5)	
	Not applicable	413 (11.5)	132 (32.0)	
	Education			<0.001
	No	3,383 (94.6)	597 (17.6)	
	Yes	38 (1.1)	21 (55.3)	
	Not applicable	155 (4.3)	68 (43.9)	
	Home			<0.001
	No	3,470 (97.0)	631 (18.2)	
	Yes	75 (2.1)	46 (61.3)	
	Not applicable	31 (0.9)	9 (29.0)	
	Social activities			<0.001
	No	3,272 (91.5)	508 (15.5)	
	Yes	282 (7.9)	171 (60.6)	
	Not applicable	22 (0.6)	7 (31.8)	

Values are presented as number (%).

1 The chi-square test comparing the prevalence of discriminatory experiences in getting hired across different categories for each predictor.

**Table 2.** Gender differences in under-reporting hiring discrimination based on the random forest prediction

Variables	Total (n)	Prevalence of hiring discrimination, n (%)	Prevalence ratio (95% CI)
Training sample (“yes” or “no” group)	3,479	686 (19.7)^1^	2.98 (2.49, 3.57)
Prediction sample (“NA” group)	97	57 (58.8)^2^	
Men (n=2,165)			
Training sample	2,101	395 (18.8)^1^	2.41 (1.82, 3.20)
Prediction sample	64	29 (45.3)^2^	
Women (n=1,411)			
Training sample	1,378	291 (21.1)^1^	4.02 (3.37, 4.79)
Prediction sample	33	28 (84.8)^2^	

NA, not available; CI, confidence interval.

1 Observed value.

2 Predicted value.

## SUPPLEMENTARY MATERIALS

Supplementary Material 1. Machine learning algorithms and tuning parameters for each model

Supplementary Material 2 Overview of the sensitivity analyses.

Supplementary Material 3. Receiver operating characteristic (ROC) curve of the cross-validated classification performance of each model

Supplementary Material 4. Variable importance scores for the predictors in the best-performing machine learning algorithm (random forest)

Supplementary Material 5. Predictive performance of the best-performing machine learning algorithm (random forest) across different probability threshold values, and the optimal value of probability threshold for classification

Supplementary Material 6. Predictive performance of the best-performing machine learning algorithm (random forest) across different probability threshold values, and the optimal value of the probability threshold for classification in the sensitivity analyses

Supplementary Material 7. Gender difference in under-reporting hiring discrimination based on the random forest prediction from sensitivity analysis 1

Supplementary Material 8. Cross-validated performance of the machine learning algorithms according to the area under the curve (AUC) in sensitivity analyses 2 and 3. CV, cross-validation; CI, confidence interval

Supplementary Material 9. Receiver operating characteristic (ROC) curve of cross-validated classification performance of each model in sensitivity analyses 2 and 3

Supplementary Material 10. Gender difference in under-reporting hiring discrimination based on the random forest prediction from sensitivity analysis 2

Supplementary Material 11. Gender difference in under-reporting hiring discrimination based on the random forest prediction from sensitivity analysis 3

Supplementary Material 12. Association between the experience of hiring discrimination and poor self-rated health among wage workers in South Korea
