# Supplementary material for: Gender differences in under-reporting hiring discrimination in Korea: a machine learning approach
Source: Epidemiol Health. 2021 Nov 17;43:e2021099. doi: 10.4178/epih.e2021099 (PMC8920741; doi:10.4178/epih.e2021099)
Supplement: Supplementary Material 1. — Machine learning algorithms and tuning parameters for each model [file epih-43-e2021099-suppl1.docx]

Supplementary Material 1. Machine learning algorithms and tuning parameters for each model

| Algorithms | Tuning parameters |
| --- | --- |
| Logistic regression |  |
| Random Forest | mtry (the number of predictors that will be randomly sampled at each split when creating the tree models),  min_n (the minimum number of data points in a node that is required for the node to be split further) |
| K-nearest neighbor | neighbors (the number of neighbors to consider),  weight_func (the type of kernel function used to weight distances between samples),  dist_power (the parameter used in calculating Minkowski distance) |
| Ridge regression | penalty (the total amount of regularization) |
| Lasso regression | penalty (the total amount of regularization) |
| Elastic net | penalty (the total amount of regularization),  mixture (the proportion of lasso regularization in the model) |
| Support vector machine  with radial | cost (the cost of predicting a sample within or on the wrong side of the margin), rbf_sigma (a positive number for radial basis function),  margin (epsilon in the SVM insensitive loss function) |
| Support vector machine  with polynomial | cost (cost of predicting a sample within or on the wrong side of the margin),  scale_factor (polynomial scaling factor),  margin (epsilon in the SVM insensitive loss function),  degree (polynomial degree) |
| Neural networks | hidden_units (the number of units in the hidden model.),  dropout (the proportion of model parameters randomly set to zero during model training),  penalty (the amount of weight decay) |
