# Supplementary material for: Gender differences in under-reporting hiring discrimination in Korea: a machine learning approach
Source: Epidemiol Health. 2021 Nov 17;43:e2021099. doi: 10.4178/epih.e2021099 (PMC8920741; doi:10.4178/epih.e2021099)
Supplement: Supplementary Material 2 — Overview of the sensitivity analyses. [file epih-43-e2021099-suppl2.docx]

Supplementary Material 2. Overview of the sensitivity analyses

| Analysis | Study population | | Probability threshold for classification | |
| --- | --- | --- | --- | --- |
|  | N | Inclusion criteria | Value | Selection criteria |
| Main analysis | 3576 | Wage workers in the seventh wave of KLIPS who had complete information for all predictors except those who answered “not applicable” to all questions about the experience of discrimination | 0.267858 | Maximizing the sum of sensitivity and specificity |
| Sensitivity analysis 1 | 3576 |  | 0.1758445 | Minimizing the difference between sensitivity and specificity |
| Sensitivity analysis 2 | 3636 | Wage workers in the seventh wave of KLIPS who had complete information for all predictors | 0.2800794 | Maximizing the sum of sensitivity and specificity |
| Sensitivity analysis 3 | 3636 |  | 0.1757411 | Minimizing the difference between sensitivity and specificity |
