# Supplementary material for: Gender differences in under-reporting hiring discrimination in Korea: a machine learning approach
Source: Epidemiol Health. 2021 Nov 17;43:e2021099. doi: 10.4178/epih.e2021099 (PMC8920741; doi:10.4178/epih.e2021099)
Supplement: Supplementary Material 4. — Variable importance scores for the predictors in the best-performing machine learning algorithm (random forest) [file epih-43-e2021099-suppl4.docx]

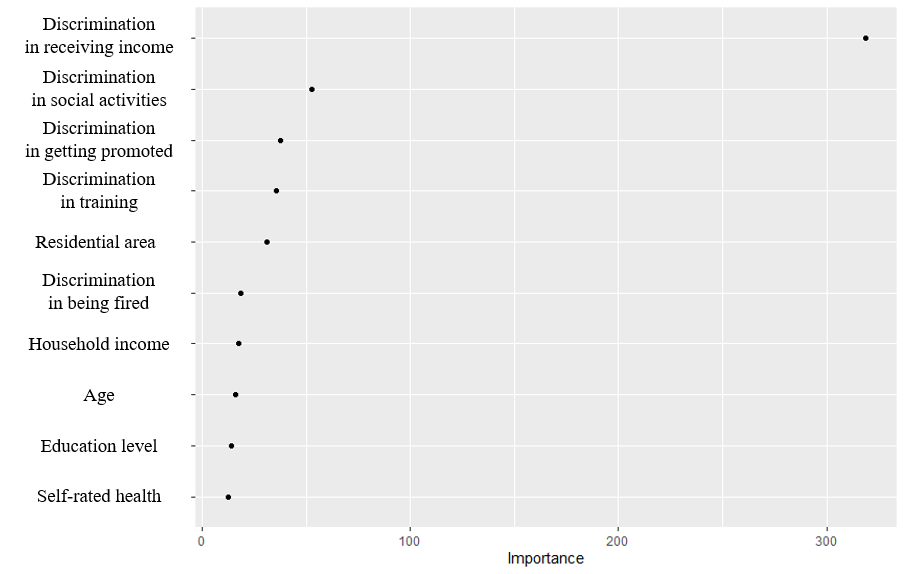


Supplementary Material 4. Variable importance scores for the predictors in the best-performing machine learning algorithm (random forest).

|  |
| --- |
