# Supplementary material for: Gender differences in under-reporting hiring discrimination in Korea: a machine learning approach
Source: Epidemiol Health. 2021 Nov 17;43:e2021099. doi: 10.4178/epih.e2021099 (PMC8920741; doi:10.4178/epih.e2021099)
Supplement: Supplementary Material 5. — Predictive performance of the best-performing machine learning algorithm (random forest) across different probability threshold values, and the optimal value of probability threshold for classification [file epih-43-e2021099-suppl5.docx]

|  |
| --- |


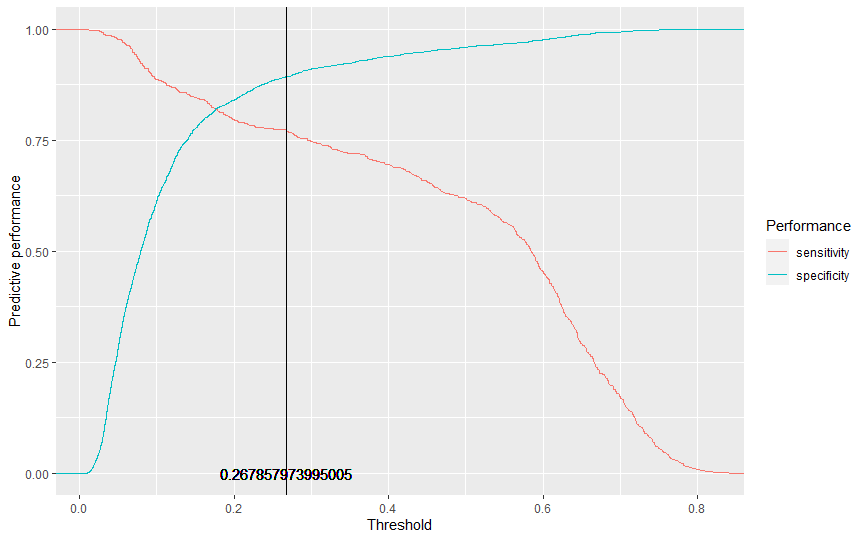


Supplementary Material 5. Predictive performance of the best-performing machine learning algorithm (random forest) across different probability threshold values, and the optimal value of probability threshold for classification.
