# Supplementary material for: Gender differences in under-reporting hiring discrimination in Korea: a machine learning approach
Source: Epidemiol Health. 2021 Nov 17;43:e2021099. doi: 10.4178/epih.e2021099 (PMC8920741; doi:10.4178/epih.e2021099)
Supplement: Supplementary Material 7. — Gender difference in under-reporting hiring discrimination based on the random forest prediction from sensitivity analysis 1 [file epih-43-e2021099-suppl7.docx]

Supplementary Material 7. Gender difference in under-reporting hiring discrimination based on the random forest prediction from sensitivity analysis 1

|  | Total | Prevalence of  hiring discrimination | Prevalence ratio (95% CI) |
| --- | --- | --- | --- |
|  | N | N (%) |  |
| Training sample (“yes” or “no” group) | 3,479 | 686 (19.7)* | 3.29 (2.80–3.87) |
| Prediction sample (“NA” group) | 97 | 63 (64.9)** |  |
| Male (n=2,165) |  |  |  |
| Training sample | 2,101 | 395 (18.8)* | 2.74 (2.13–3.53) |
| Prediction sample | 64 | 33 (51.6)** |  |
| Female (n=1,411) |  |  |  |
| Training sample | 1,378 | 291 (21.1)* | 4.30 (3.71–4.99) |
| Prediction sample | 33 | 30 (90.9)** |  |

*Observed value, **Predicted value. NA, not applicable.

NA, not available; CI, confidence interval.
