# Supplementary material for: Gender differences in under-reporting hiring discrimination in Korea: a machine learning approach
Source: Epidemiol Health. 2021 Nov 17;43:e2021099. doi: 10.4178/epih.e2021099 (PMC8920741; doi:10.4178/epih.e2021099)
Supplement: Supplementary Material 8. — Cross-validated performance of the machine learning algorithms according to the area under the curve (AUC) in sensitivity analyses 2 and 3. CV, cross-validation; CI, confidence interval [file epih-43-e2021099-suppl8.docx]

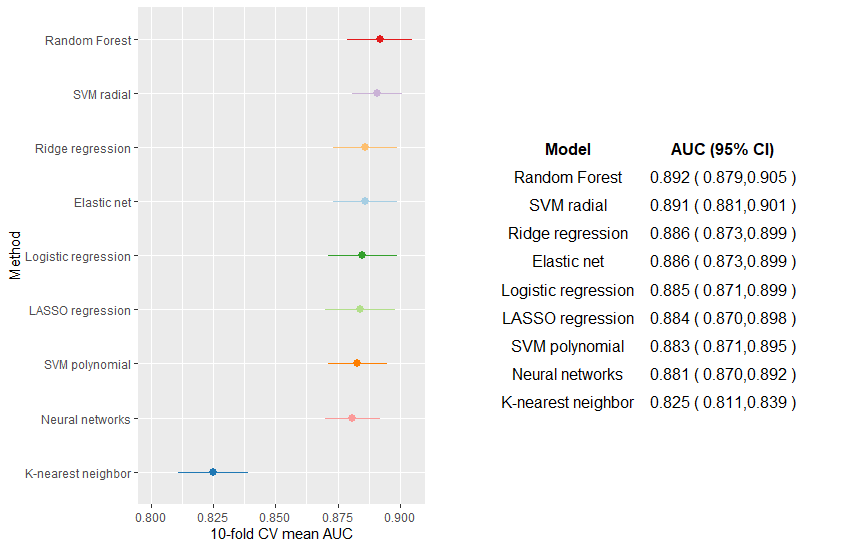


Supplementary Material 8. Cross-validated performance of the machine learning algorithms according to the area under the curve (AUC) in sensitivity analyses 2 and 3. CV, cross-validation; CI, confidence interval.
