# Supplementary material for: Gender differences in under-reporting hiring discrimination in Korea: a machine learning approach
Source: Epidemiol Health. 2021 Nov 17;43:e2021099. doi: 10.4178/epih.e2021099 (PMC8920741; doi:10.4178/epih.e2021099)
Supplement: Supplementary Material 9. — Receiver operating characteristic (ROC) curve of cross-validated classification performance of each model in sensitivity analyses 2 and 3 [file epih-43-e2021099-suppl9.docx]

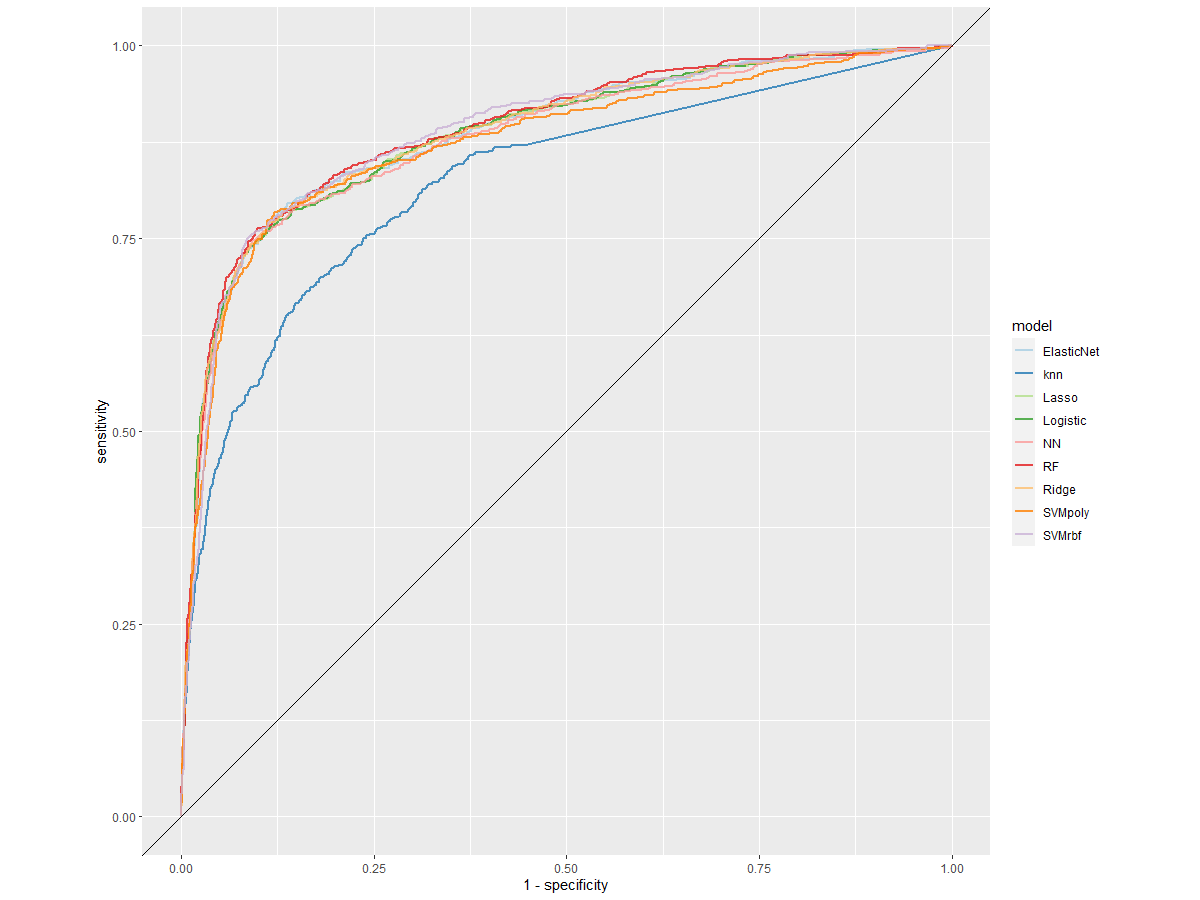


Supplementary Material 9. Receiver operating characteristic (ROC) curve of cross-validated classification performance of each model in sensitivity analyses 2 and 3.
