# Supplementary material for: Gender differences in under-reporting hiring discrimination in Korea: a machine learning approach
Source: Epidemiol Health. 2021 Nov 17;43:e2021099. doi: 10.4178/epih.e2021099 (PMC8920741; doi:10.4178/epih.e2021099)
Supplement: Supplementary Material 10. — Gender difference in under-reporting hiring discrimination based on the random forest prediction from sensitivity analysis 2 [file epih-43-e2021099-suppl10.docx]

Supplementary Material 10. Table S4. Gender difference in under-reporting hiring discrimination based on the random forest prediction from sensitivity analysis 2

|  | Total | Prevalence of  hiring discrimination | Prevalence ratio (95% CI) |
| --- | --- | --- | --- |
|  | N | N (%) |  |
| Training sample (“yes” or “no” group) | 3,482 | 688 (19.8)* | 3.75 (3.34–4.20) |
| Prediction sample (“NA” group) | 154 | 114 (74.0)** |  |
| Male (n=2,196) |  |  |  |
| Training sample | 2,103 | 397 (18.9)* | 3.30 (2.76–3.96) |
| Prediction sample | 93 | 58 (62.4)** |  |
| Female (n=1,440) |  |  |  |
| Training sample | 1,379 | 291 (21.1)* | 4.35 (3.83–4.94) |
| Prediction sample | 61 | 56 (91.8)** |  |

*Observed value, **Predicted value.

NA, not available; CI, confidence interval.
