# Supplementary material for: Gender differences in under-reporting hiring discrimination in Korea: a machine learning approach
Source: Epidemiol Health. 2021 Nov 17;43:e2021099. doi: 10.4178/epih.e2021099 (PMC8920741; doi:10.4178/epih.e2021099)
Supplement: Supplementary Material 12. — Association between the experience of hiring discrimination and poor self-rated health among wage workers in South Korea [file epih-43-e2021099-suppl12.docx]

Supplementary Material 12. Association between the experience of hiring discrimination and poor self-rated health among wage workers in South Korea (n=3,576)

| Experience of  hiring discrimination | Distribution | Prevalence of  poor self-rated health | Prevalence ratio | 95% CI |
| --- | --- | --- | --- | --- |
|  | N (%) | N (%) |  |  |
| Total (n=3,576) |  |  |  |  |
| No | 2,793 (78.1) | 156 (5.6) | *Reference* |  |
| Yes | 686 (19.2) | 70 (10.2) | 1.83 | 1.40–2.39 |
| NA-no | 40 (1.1) | 4 (10.0) | 1.79 | 0.70–4.59 |
| NA-yes | 57 (1.6) | 9 (15.8) | 2.83 | 1.52–5.25 |
| Male (n=2,165) |  |  |  |  |
| No | 1,706 (78.8) | 86 (5.0) | *Reference* |  |
| Yes | 395 (18.2) | 32 (8.1) | 1.61 | 1.09–2.38 |
| NA-no | 35 (1.6) | 3 (8.6) | 1.70 | 0.57–5.12 |
| NA-yes | 29 (1.3) | 3 (10.3) | 2.05 | 0.69–6.11 |
| Female (n=1,411) |  |  |  |  |
| No | 1,087 (77.0) | 70 (6.4) | *Reference* |  |
| Yes | 291 (20.6) | 38 (13.1) | 2.03 | 1.40–2.94 |
| NA-no | 5 (0.4) | 1 (20.0) | 3.11 | 0.53–18.19 |
| NA-yes | 28 (2.0) | 6 (21.4) | 3.33 | 1.58–7.01 |
| We examined the association between hiring discrimination and self-rated health by applying a modified Poisson regression model with a robust error variance after dividing the response to hiring discrimination among the entire population into 4 groups.  CI, confidence interval. | | | | |
